# Mechanism of action and potential applications of selective inhibition of microsomal prostaglandin E synthase-1-mediated PGE_2_ biosynthesis by sonlicromanol’s metabolite KH176m

**DOI:** 10.1038/s41598-020-79466-w

**Published:** 2021-01-13

**Authors:** X. Jiang, H. Renkema, B. Pennings, S. Pecheritsyna, J. C. Schoeman, T. Hankemeier, J. Smeitink, J. Beyrath

**Affiliations:** 1 Khondrion BV, Nijmegen, The Netherlands; 2 Department of Pediatrics, RCMM, RadboudUMC, Nijmegen, The Netherlands; 3 Faculty of Science, Leiden Academic Centre for Drug Research, Analytical BioSciences, Einsteinweg 55, 2333 CC Leiden, The Netherlands

**Keywords:** Cell signalling, Drug regulation, Pharmacology, Target identification, Infectious diseases, Cell biology, Drug discovery, Diseases

## Abstract

Increased prostaglandin E2 (PGE_2_) levels were detected in mitochondrial disease patient cells harboring nuclear gene mutations in structural subunits of complex I, using a metabolomics screening approach. The increased levels of this principal inflammation mediator normalized following exposure of KH176m, an active redox-modulator metabolite of sonlicromanol (KH176). We next demonstrated that KH176m selectively inhibited lipopolysaccharide (LPS) or interleukin-1β (IL-1β)-induced PGE_2_ production in control skin fibroblasts. Comparable results were obtained in the mouse macrophage-like cell line RAW264.7. KH176m selectively inhibited mPGES-1 activity, as well as the inflammation-induced expression of mPGES-1. Finally, we showed that the effect of KH176m on mPGES-1 expression is due to the inhibition of a PGE_2_-driven positive feedback control-loop of mPGES-1 transcriptional regulation. Based on the results obtained we discuss potential new therapeutic applications of KH176m and its clinical stage parent drug candidate sonlicromanol in mitochondrial disease and beyond.

## Introduction

Sonlicromanol (also known as KH176), a clinical-stage oral drug candidate, has been developed to combat mitochondrial disease. Previously we reported that this active parent compound and its in vivo active metabolite KH176m act as potent ROS-redox modulators^1–4^.

In order to further characterize the redox pathology in mitochondrial complex I deficient patient-derived cells, and examine the effect of our compounds in these cells, we have applied a novel metabolomics-based screening method targeted at inflammatory, oxidative and nitrosative stress markers, allowing for the exploration of the role of oxidative stress and signaling lipids^5^. We found that levels of five interlinked prostaglandins (PG) were significantly increased in primary human skin fibroblasts (PHSF) from patients with complex I deficiencies, compared with healthy control cells. Interestingly, we also found that KH176m could selectively decrease the level of the prostaglandin E2 (PGE_2_).

**Figure 1:**
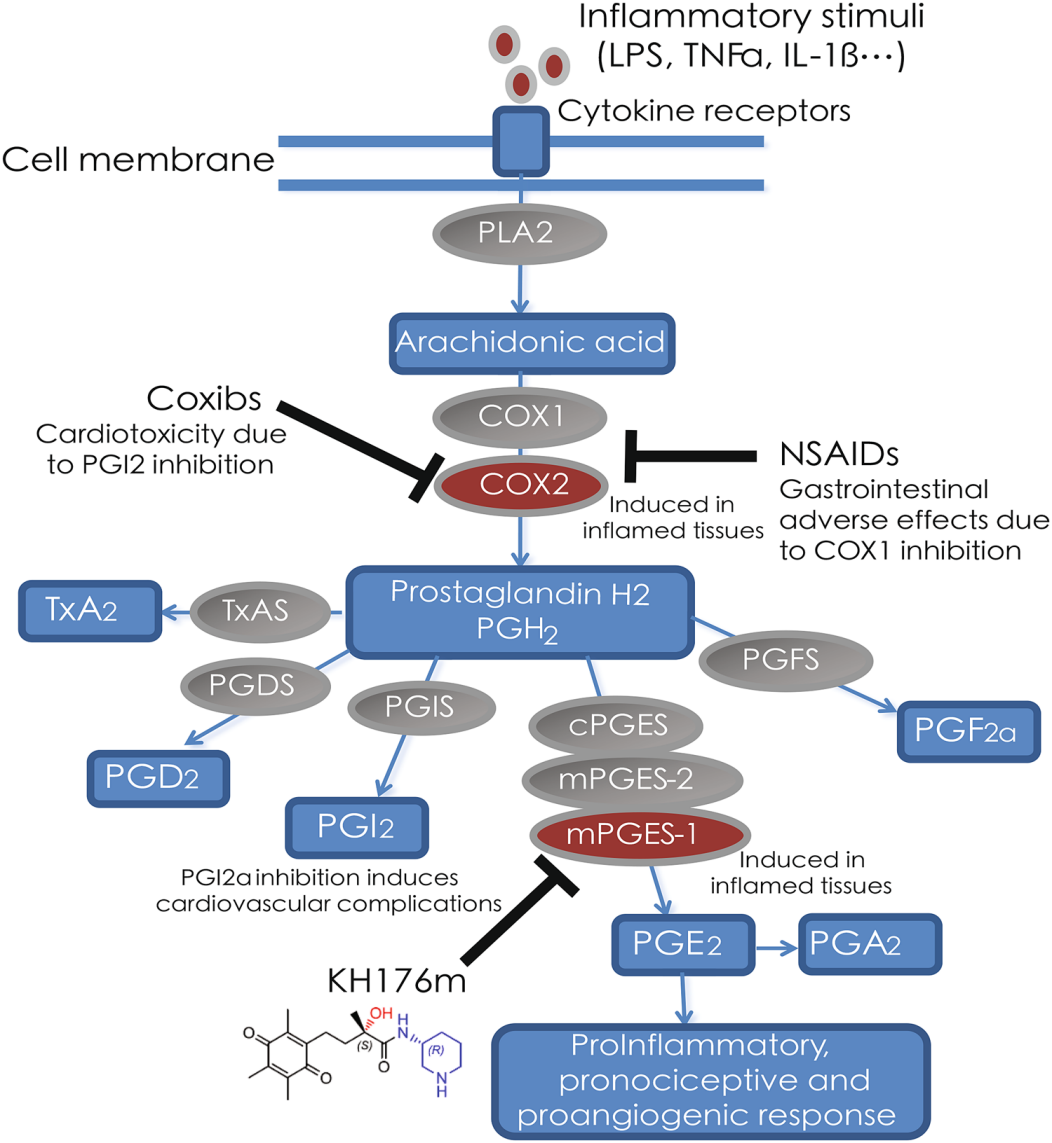
Synthesis pathways of prostaglandins and drug targeting strategies. Involved enzymes are indicated with oval shapes, PGs and intermediates with boxes.

PGs are important lipid mediators that sustain physiological and homeostatic functions but can also induce pathologic responses such as inflammatory and nociceptive responses^6^. Prostaglandins are synthesized from arachidonic acid (AA), which is released from the cell membrane by phospholipase A2 (PLA_2_). Cyclooxygenase isoforms 1 and 2 (COX-1 and COX-2) enzymes metabolize AA into prostaglandin G2 (PGG_2_) and subsequently to prostaglandin H2 (PGH_2_) by bis-oxygenation and peroxidation reactions, respectively. PGH_2_ is the common precursor of the four principal bioactive prostaglandins PGD_2_, PGI_2_, PGE_2_, and PGF_2α_ and the prostanoid thromboxane A2 (TXA_2_) that are synthetized by cell- and tissue-specific synthases and isomerases (Fig. 1)^7–9^.

PG levels are commonly elevated in inflamed tissues and are known to induce and propagate the inflammation response^10^. Among the prostanoids, PGE_2_ has the greatest impact on the processing of inflammatory pain signals^11^. PGE_2_ is synthesized from PGH_2_ by three different PGE_2_ synthases which are either membranous (mPGES-1, mPGES-2) or cytosolic (cPGES) enzymes^12^. Of these PGE_2_ synthases, cPGES and mPGES-2 are constitutively expressed in many organs and tissues, whereas mPGES-1, like COX-2, is up-regulated in response to various inflammatory stimuli^13–15^. PGE_2_ has been shown to enhance the transcriptional expression of mPGES-1 in combination with inflammatory stimuli revealing a PGE_2_-mediated positive feedback control loop of the product on its own enzyme. Finally, mPGES-1 has also been shown to be selectively increased in several types of cancer and is associated with poor prognoses^7,16,17^.

mPGES-1 has recently gained attention as a safer target for anti-inflammatory drugs since it is solely expressed in diseased tissue and downstream of the COX enzymes. Though COX enzymes are the current target for most commercially available non-steroidal anti-inflammatory drugs (NSAIDs), their inhibition leads to the unspecific decrease of major PGs and their use can be limited because of gastric side effects or increased risk of cardiovascular morbidity and mortality.

Based on our screening results, we further investigated the effect of KH176m on PGE_2_ biosynthesis in human control primary fibroblast cells, as well as in the mouse macrophage-like cell line RAW264.7. Our data indicates that KH176m could selectively block the production of PGE_2_ induced by the inflammatory stimuli lipopolysaccharide (LPS) or interleukin-1 beta (IL-1β) in both cell types, without affecting the levels of other prostaglandins. We further demonstrated that the inhibitory effect of KH176m on PGE_2_ production is dependent on mPGES-1 inhibition, further blocking mPGES-1 transcriptional expression. Therefore, in addition to be a novel therapeutic option for mitochondrial disease patients, our results indicate that KH176m as well as its parent compound sonlicromanol may also potentially be used to treat PGE_2_-driven inflammatory consequences such as inflammatory pain or cancer.

## Results

### KH176m selectively decreases the elevated level of PGE_2_ in primary human skin fibroblasts from patients with mitochondrial disease

Based on our previous work, sonlicromanol, and its in vivo active metabolite KH176m, were both identified as potent ROS-redox modulators. Sonlicromanol is currently in clinical development for patients with mitochondrial disease^14^. To extend the phenotypical analysis of complex I deficient mitochondrial disease (MD) primary fibroblasts, we analyzed these cells and the supernatants with a new LC–MS metabolomics-based method for oxidative, nitrosative, and inflammatory stress^5^.

**Figure 2:**
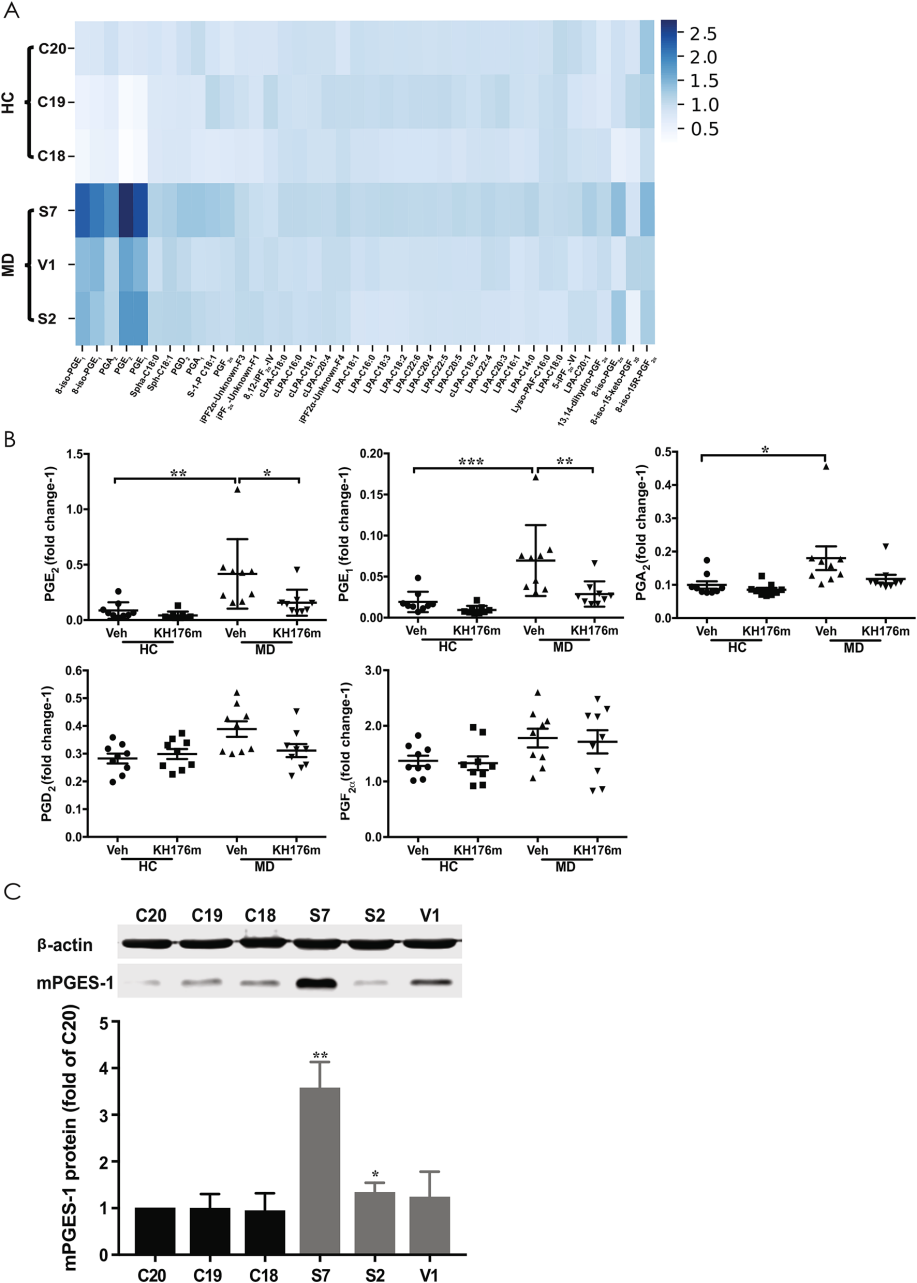
KH176m selectively decreases elevated PGE_2_ in primary human skin fibroblasts from patients with mitochondrial disease. (**A**) Heat map showing the extracellular levels of oxidative stress related metabolites from three healthy control (HC) (C20, C19, C18) and three primary mitochondrial disease cell lines (MD) (S7, S2, V1) with complex I deficiency. Cells were analyzed on the oxidative stress platform. Metabolites are plotted on x-axis, and the cell lines on the y-axis. The blue color indicates high relative levels, and white indicates low relative levels. Heat map is produced with seaborn package (version: 0.11.0) in python (URL: https://doi.org/10.5281/zenodo.592845)^47^. (**B**) Quantitative average of PGA_2_, PGD_2_, PGE_1_, PGE_2_, and, PGF_2α_ in HC or MD cells exposed to 1 µM KH176m or vehicle for 24 h is shown. Bar graphs represent the average of 3 independent measurements ± SD (n = 9). The comparisons between multiple groups were determined by analysis of variance (ANOVA) for parametric data. *p < 0.05; significant difference compared with vehicles. (**C**) Total protein of three HC (C20, C19, C18) and three MD (S7, S2, V1) cell lines with complex I deficiency were extracted and separated by SDS-PAGE, and expression of mPGES-1 was analyzed by western blot. (**D**) Quantification of the western blot analysis for mPGES-1. Bar graphs represent the average of at least 3 independent measurements ± SD, and are normalized on the vehicle condition. (n = 4). *p < 0.05; **p < 0.005; significant difference compared with C20.

As shown in Fig. 2A, three fibroblast cell lines from healthy volunteers (C5120, C5119, and C5118) and three fibroblast cell lines from mitochondrial Complex I deficient (MD) patients [S7-5175 (NdufS7, V112M mutation), S2-7277 (NdufS2, R228Q mutation), and V1-5171 (NdufV1, R59X/T423M mutation)] were exposed for 24 h to 1 µM KH176m or vehicle, after which the cells were processed for metabolomics analysis. We observed that five interconnected inflammatory biomarker prostaglandins (PGA_2_, PGE_1_, PGE_2_, 8-iso-PGE_1_, and 8-iso-PGE_2_) were significantly increased in MD fibroblasts, compared with healthy control cells (Fig. 2A). We did not detect significant changes in any of the other 40 assessed metabolites (Fig. 2A). Furthermore, our data also showed that elevated PGE_1_, and PGE_2_ levels were significantly decreased by treatment with 1 µM KH176m for 24 h (Fig. 2A,B).

PGA_2_ is produced by PGE_2_ following rapid non-enzymatic dehydration. PGE_1_ is derived from omega 6 fatty acids, and acts via the PGE_2_ receptor. PGE_1_ metabolites play an important role in the balancing act between PG groups to manage inflammation, with a primary anti-inflammatory effect on the tissue microenvironment^18^. The 8-iso-PGE_1_ is a large scale biosynthetic production of PGE_1_ from eicosatrienoic acid^19^. The 8-iso-PGE_2_ is produced from arachidonic acid during lipid peroxidation and has been identified as metabolites of PGE_2_^19,20^. Interestingly, the levels of other prostaglandins, such as PGD_2_ and PGF_2α_, were not significantly affected by mitochondrial disease as well as KH176m exposure (Fig. 2A,B). Using this same panel of fibroblasts we studied the expression of the induced enzymes involved in the synthesis of PGE_2_ using Western-blot analysis of steady state grown cells. COX-2, an enzyme responsible for induced prostaglandin (PG) biosynthesis, was found to be below the level of detection, however the mPGES-1 protein levels were increased in 2 out of 3 MD cell lines (up to 3.5-fold) (Fig. 2C,D).

**Figure 3:**
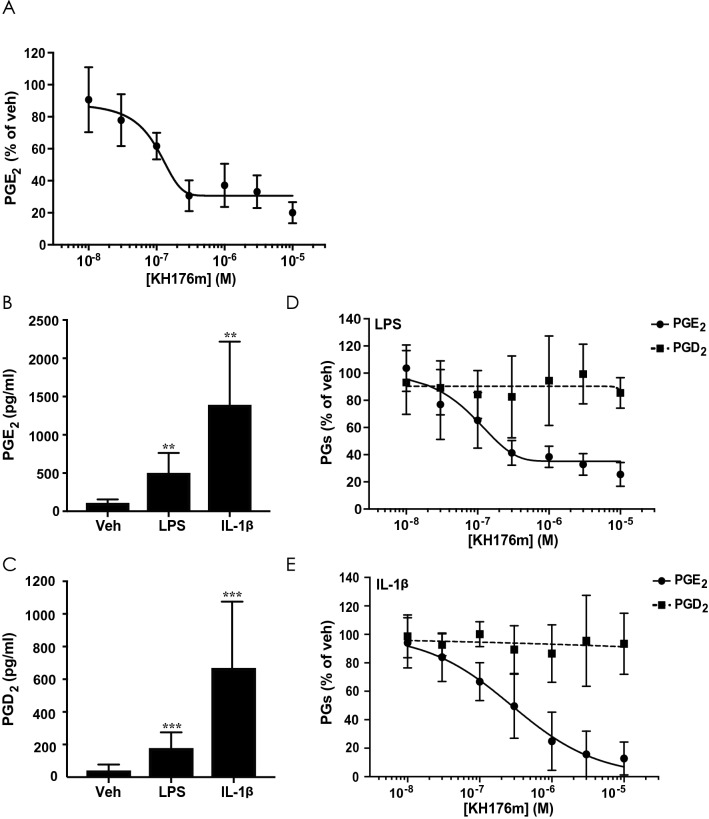
KH176m selectively and dose-dependently inhibits PGE_2_ induced by inflammatory stimuli LPS or IL-1β in primary human skin fibroblasts. (**A**) Level of PGE_2_ was analyzed in the culture medium of fibroblasts after treatment with KH176m for 72 h (n = 4). (**B**) Levels of PGE_2_ and PGD_2_ were analyzed in the culture medium of fibroblasts stimulated with LPS (1 µg/mL) or (**C**) IL-1β (1 ng/mL) for 24 h. Bar graphs represent the average of at least 3 independent measurements ± SD (n = 6–8). (**D**) Levels of PGE_2_ and PGD_2_ in the culture medium of fibroblasts stimulated with (**D**) LPS (1 µg/mL) or (**E**) IL-1β (1 ng/mL) for 24 h alone (set as 100%) or in combination with increasing concentrations of KH176m (n = 3). **p < 0.005; ***p < 0.001; significant differences compared with vehicles.

We confirmed the effect of KH176m on PGE_2_ using an ELISA method, in which fibroblasts were treated with increasing concentrations of KH176m for 72 h, and levels of PGE_2_ were quantified in the cell supernatant. This longer incubation time was required to compensate for the lower sensitivity of the ELISA method. KH176m was found to dose-dependently decrease PGE_2_ levels, with an IC_50_ value of 85.3 ± 17.8 nM (Fig. 3A).

Since PGE_2_ synthesis is increased following inflammatory stimuli, we evaluated the effect of KH176m on LPS- or IL-1β -induced levels of PGE_2_. As a control for selectivity, we also studied changes in PGD_2_ production. As expected, after 24 h incubation of control fibroblasts with either LPS or IL-1β, PGE_2_ and PGD_2_ levels in the supernatants of the cells were significantly increased (Fig. 3B,C). KH176m treatment efficiently reduced PGE_2_ levels, but not other PGs, with IC_50_ 92.9 ± 23.5 nM or 0.28 µM, in supernatant of cells treated with LPS (Fig. 3D) or IL-1β (Fig. 3E, Supplemental Figure 1A and B), respectively. Of note, in our experimental conditions IL-1β led to an approximate four-fold higher increase in PGE_2_ production as compared with LPS, which might explain the differences in KH176m potencies following the different stimulations. Thus, production of PGE_2_, a well-known inflammatory mediator, was selectively blocked in the presence of KH176m in human fibroblasts treated with inflammatory stimuli.

### KH176m selectively and dose-dependently decreases the level of PGE_2_ in RAW264.7 macrophage-like cells

**Figure 4:**
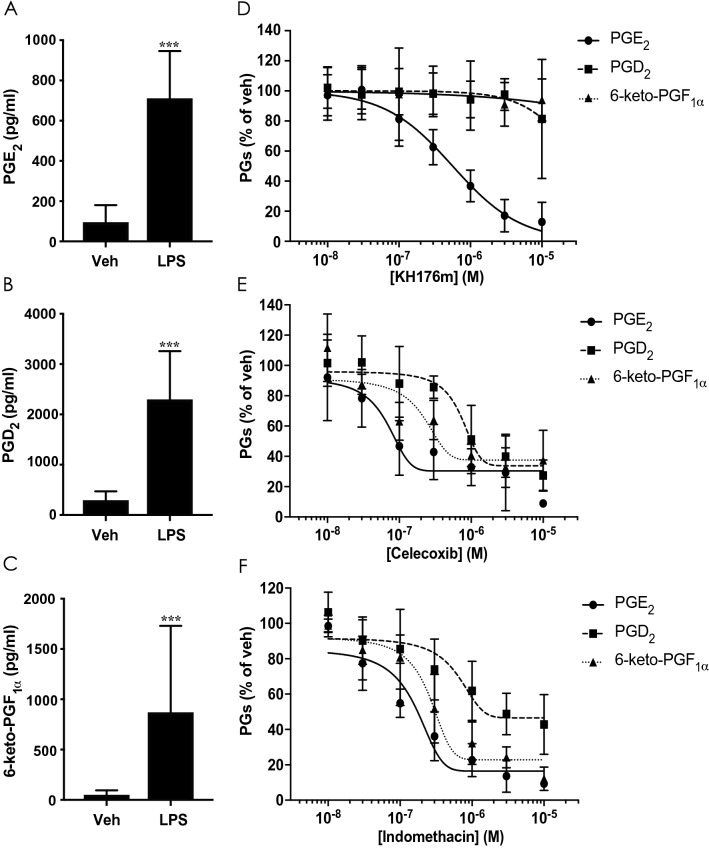
KH176m selectively and dose-dependently decreases the level of PGE_2_ induced by LPS in mouse macrophage-like cell RAW264.7. Levels of (**A**) PGE_2_, (**B**) PGD_2_, and (**C**) 6-keto-PGF_1α_ were analyzed in the culture medium of RAW264.7 cells stimulated with vehicle or LPS (1 µg/mL) for 24 h. Bar graphs represent the average of at least 3 independent measurements ± SD (n = 6–8). Levels of PGE_2_, PGD_2_, and 6-keto-PGF_1α_ in the culture medium of RAW264.7 stimulated with LPS (1 µg/mL) alone or in combination with increasing concentrations of KH176m (**D**), celecoxib (**E**), or indomethacin (**F**) (LPS alone set as 100%) (n = 3). ***p < 0.001; significant difference compared with vehicles.

LPS is a well-known and powerful macrophage activator. LPS treated RAW264.7 cells are a defined model of macrophage activation at the site of inflammation^21^. During inflammation, macrophages are a central source of PGE_2_ production. Therefore, we used an LPS-induced macrophage cell model (RAW264.7) to investigate the effect of KH176m on prostaglandin levels. Cells were treated with the inflammatory stimulus LPS alone or in combination with increasing concentrations of KH176m. The COX-2 inhibitor celecoxib or the COX-1/2 inhibitor indomethacin was used as controls. After 24 h incubation, the culture medium levels of PGE_2_, PGD_2_ and 6-keto-PGF_1α_ (a stable metabolite of PGI_2_ commonly measured as a surrogate of PGI_2_), were quantified by ELISA. As expected, LPS or IL-1β efficiently induced PGs production (Fig. 4A–C, Supplemental Figure 1C and D), with KH176m dose-dependently and selectively reducing the level of PGE_2_ (IC_50_ 0.56 ± 0.08 µM), showing no effect on the other two prostaglandins (Fig. 4D, Supplemental Figure 1E). While all tested PGs could be reduced in a dose-dependent manner via exposure to the COX inhibitor celecoxib or indomethacin (Fig. 4E,F). The specific effect of KH176m on the production of PGE_2_ was therefore confirmed in this LPS-induced acute inflammation cell model. It was reported that selective inhibition of mPGES-1 may shunt its substrate PGH_2_ to increase the level other prostaglandins, since COX-2 activity is increased under inflammatory status^22^. It is important to note that in our experiments, KH176m treatment of RAW264.7 cells did not affect PGD_2_ levels_,_ or 6-keto-PGF_1α_ production, when PGE_2_ levels were decreased (Supplemental Figure 2A). KH176i, a redox-inactive form of KH176 which was produced by substituting the hydroxyl function within the chromanyl group by a methoxy moiety as expected was unable to reduce PGE_2_ level (Supplemental Figure 2B).

### KH176m inhibits mPGES-1 enzyme activity and transcription

**Figure 5:**
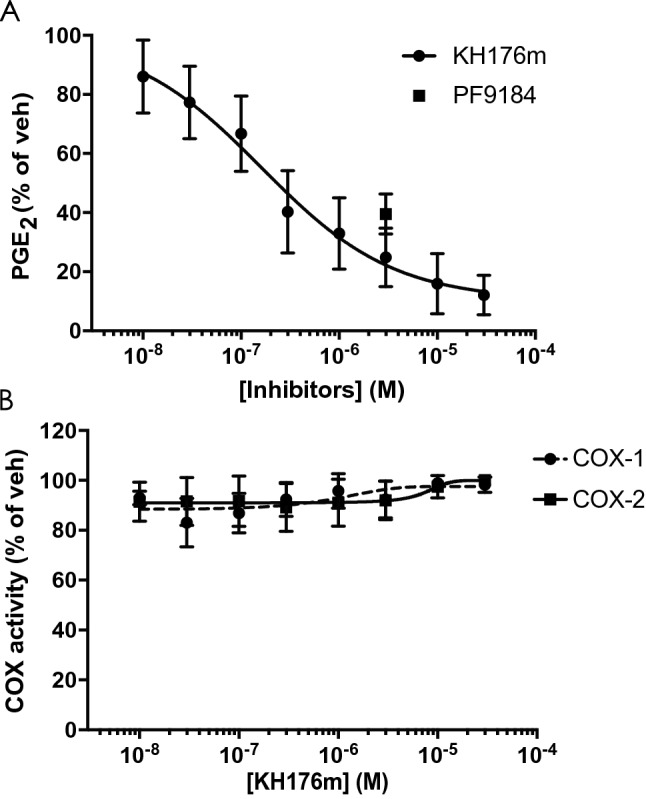
KH176m decreases the activity of mPGES-1 enzyme. RAW264.7 cells were treated with LPS (1 µg/mL) for 24 h and microsomes were isolated and used as source of mPGES-1 for ex vivo inhibition experiments. (**A**) The activity of mPGES-1 was measured in microsomes fraction as the conversion of PGH_2_ to PGE_2_ (n = 6). (**B**) The activity of COX-1 and COX-2 were measured using the COX Inhibitor Screening Kit (n = 3).

Based on the selective control of PGE_2_ production by KH176m upon inflammatory stimuli, we assessed the effect of KH176m on the activity and expression of the mPGES-1 enzyme. RAW264.7 cells were treated with the inflammatory stimulus LPS to increase the expression of mPGES-1. After 24 h incubation, microsomes were isolated from the cells and exposed to increasing concentrations of KH176m or a single concentration of the previously described mPGES-1 inhibitor PF9184 for 15 min, ex vivo^23^. mPGES-1 activity was assayed in the microsome fractions by using PGH_2_ as a substrate that was converted to PGE_2_ and subsequently quantified. The results showed that the mPGES-1 enzymatic activity was inhibited in purified microsomes treated with KH176m or the positive control PF9184; the IC50 of KH176m was 0.16 ± 0.048 µM (Fig. 5A). Similar results were obtained in primary human control skin fibroblasts, and the IC50 of KH176m was 1.51 ± 0.93 µM (Supplemental Figure 3). Furthermore, KH176m had no effect on recombinant COX-1 or COX-2 enzymes activity (Fig. 5B).

**Figure 6:**
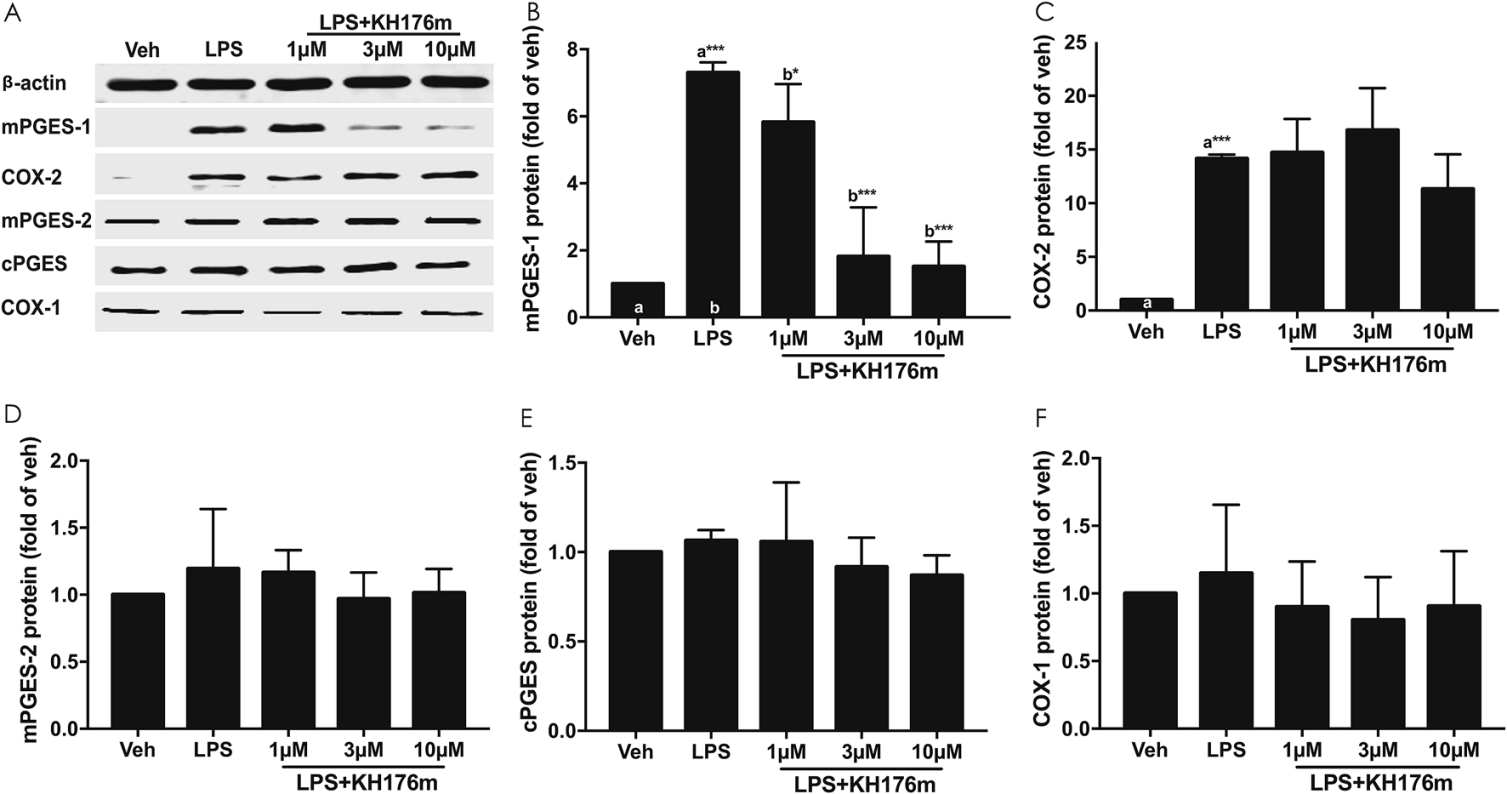
KH176m selectively decreases the expression of mPGES-1 enzyme induced by LPS. RAW264.7 cells were treated with various concentrations of KH176m in the presence of LPS (1 µg/mL) for 24 h or 6 h. (**A**) Protein was isolated and separated by SDS-PAGE, and expression of indicated proteins were analyzed by western blot. Quantification of the western blot analysis for (**B**) mPGES-1, (**C**) COX-2, (**D**) mPGES-2, (**E**) cPGES, and (**F**) COX-1. Bar graphs represent the average of at least 3 independent measurements ± SD, and are normalized on the vehicle condition. (n = 3) *p < 0.05; ***p < 0.001; significant differences compared with the marked conditions (a,b).

We investigated the expression of eicosanoid enzymes responsible for the synthesis of PGE_2_ in the RAW264.7 cell model. Cells were treated with the inflammatory stimulus LPS alone or in combination with increasing concentrations of KH176m. After 6 or 24 h incubation, mPGES-1, mPGES-2, cPGES, COX-1, and COX-2 RNA and protein levels were quantified in cells by qRT-PCR and Western-blot, respectively. As expected, LPS efficiently induced the expression of the two inducible enzymes, mPGES-1 and COX-2, at both the protein (Fig. 6A–C) and gene (Supplemental Figure 4A and B) levels. Protein (Fig. 6D–F) and mRNA (Supplemental Figure 4C–E) levels of the constitutively expressed enzymes mPGES-2, cPGES and COX-1 remained unchanged. Treatment with KH176m dose-dependently reduced LPS-induced expression of mPGES-1, but not COX-2, at the protein (Fig. 6A–C) and mRNA (Supplemental Figure 4A and B) levels. The constitutive protein and mRNA expression levels of mPGES-2, cPGES, and COX-1 remained unchanged after KH176m exposure (Fig. 6A,D–F and Supplemental Figure 4C–E). Taken together, these results showed that KH176m selectively inhibits transcriptional expression of mPGES-1 enzyme induced by the inflammatory stimulus LPS, explaining the compound’s selectivity in reducing PGE_2_ levels, but not those of other prostaglandins.

### KH176m effect on mPGES-1 transcriptional regulation is overcome by exogenous addition of PGE_2_

**Figure 7:**
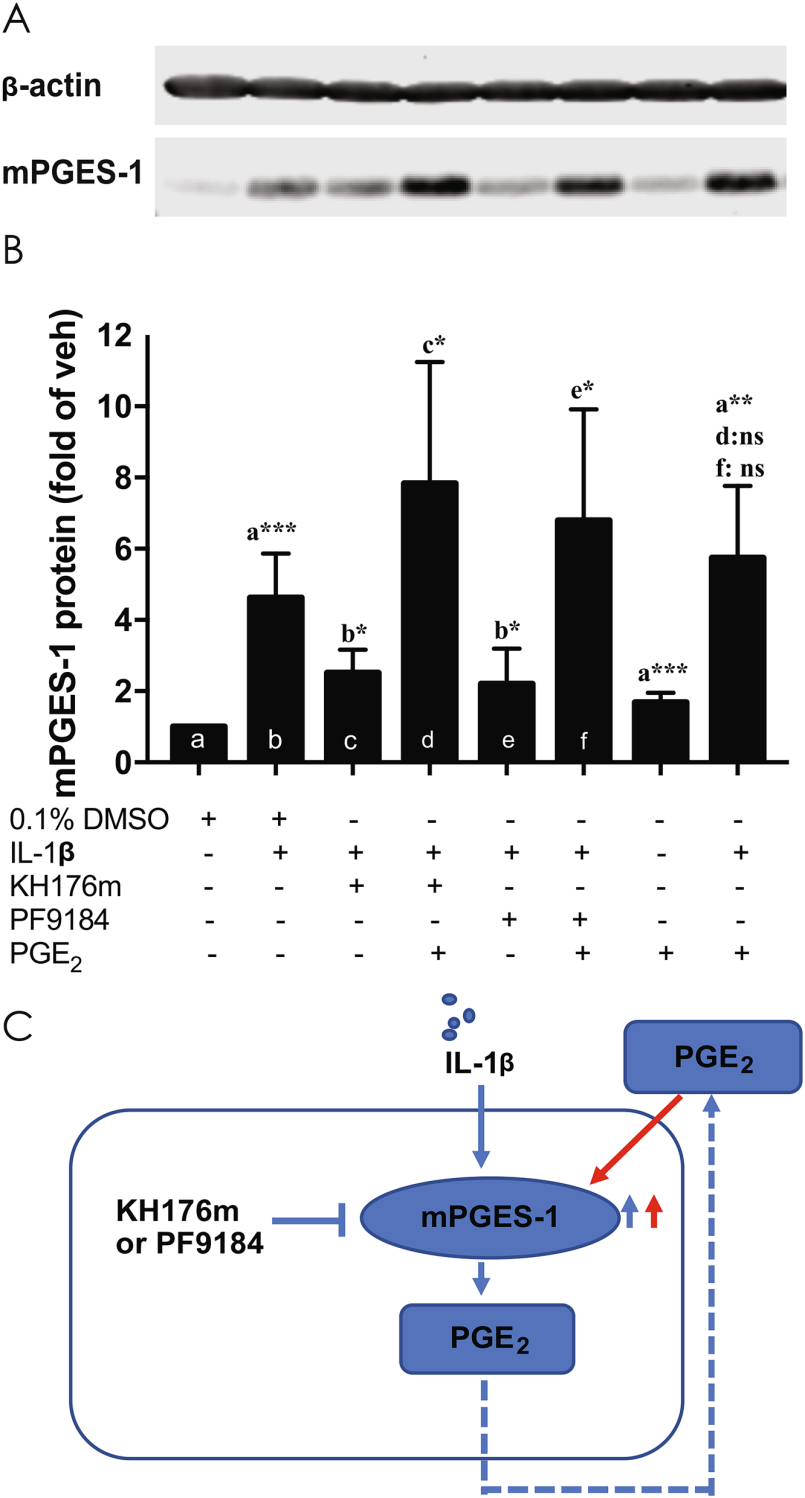
Exogenous PGE_2_ reversed the effect of KH176m in IL-1β stimulated fibroblasts. Fibroblasts were treated with KH176m (3 µM) or PF9184 (3 µM) ±  PGE_2_ (100 nM) in the presence of IL-1β (1 ng/mL). (**A**) After 24 h, protein was isolated and separated by SDS-PAGE, and expression of indicated proteins were analyzed by western blot. (**B**) Quantification of the western blot analysis. Bar graphs represent the average of at least 3 independent measurements ± SD, and are normalized on the vehicle condition. (n = 3) *p < 0.05; **p < 0.005; ***p < 0.001; significant differences compared with the marked conditions (a,b,c,d,e). (**C**) Schematic representation of the results indicating how PGE_2_ positively regulated mPGES-1 and is thereby responsible for its own biosynthesis.

As a dual effect of KH176m on both the activity and expression of mPGES-1 seemed unlikely, we sought a possible mechanism that might reconcile both of these effects. It was previously shown that increased synthesis of PGE_2_, in combination with an inflammatory stimulus, can enhance the expression of its own enzyme, mPGES-1^24^. We therefore hypothesized that KH176m inhibits mPGES-1 activity, reducing PGE_2_ production and, consequently, blocking the PGE_2_-driven positive feedback control of mPGES-1 transcriptional regulation. We assessed whether an exogenous addition of PGE_2_ could overcome the inhibitory effect of KH176m on LPS- or IL-1β-induced mPGES-1 expression. Treatment of primary human skin fibroblasts with increasing concentrations of exogenous PGE_2_ (1–200 nM) for 24 h revealed a small and dose-dependent (optimum at 100 nM of PGE_2_) increase of mPGES-1, although this effect was not as pronounced as with IL-1β treatment (Supplemental Figure 5A and B). We also measured the expression of mPGES-1 mRNA and protein after 24 h treatment with IL-1β in the presence or absence of KH176m and exogenous PGE_2_. To further test our hypothesis, we also included the known inhibitor of mPGES-1 activity, PF9184. As previously shown, IL-1β-driven increases in protein (Fig. 7A,B) and mRNA (Supplemental Figure 5C) expression of mPGES-1 were inhibited by KH176m. Interestingly, the same effect was also observed with the mPGES-1-specific inhibitor, PF9184. Under conditions of activation by IL-1β and inhibition of mPGES-1 enzyme activity by either KH176m or PF9184, the addition of exogenous PGE_2_ resulted in the restoration of high mPGES-1 protein (Fig. 7) and mRNA levels (Supplemental Figure 4). These results demonstrated that exogenous PGE_2_ treatment reversed the effect of KH176m or PF9184 in IL-1β stimulated fibroblasts, suggesting a positive feedback regulation of the PGE_2_ product on the expression of its enzyme mPGES-1, which was directly inhibited by either compound (Fig. 7C).

## Discussion

Reactive oxygen species (ROS) are the major host defense agents against infection and other toxins^25^. Within mitochondria, eleven different sites are known to produce superoxide and/or hydrogen peroxide (ROS) by leaking electrons to oxygen^26–28^. Mitochondrial Complex I (NADH:ubiquinone oxidoreductase, E.C. 1.6.5.3), when deficient like in m.3243A > G MELAS spectrum disorders, is a major source of ROS production and has been found to trigger inflammation^29^. Mitochondria can therefore act through redox-sensitive inflammatory pathways or by direct activation of the inflammasome to modulate innate immunity^30^. Previous studies have suggested that scavenging of the by-products associated with excessive oxidative stress produced by mitochondria may represent a novel therapeutic intervention for inflammation. Increased ROS levels have previously been reported in MD patient cells^3,31^. Mitochondrial dysfunction leads to mitochondrial ROS production as well as low-grade expression of COX-2^32^. The increase in COX-2 expression was accompanied by a dose-dependent increase in PGs release. Also increased intracellular ROS can stimulate the production of PGE_2_, has been found by Hu et al^33^. Importantly, we now present significant increases in PGE_2_ in complex I deficient mitochondrial disease cell lines harboring mutations in different nuclear genes encoding structural proteins of NADH:ubiquinone oxidoreductase. This was corroborated by increased expression of mPGES-1 in 2/3 patient cell lines. We are currently exploring a larger panel of mitochondrial disease patient cell lines. Treatment of these fibroblasts with the redox-modulating compound KH176m decreased the PGE_2_ release from these cells not only in a dose-dependent manner but also after activation of PGE_2_ production by the bacterial endotoxin LPS or the cytokine IL-1β. This effect was PGE_2_-specific, since PGD_2_ levels, which were also increased in the MD samples, were not decreased by exposure to the compound.

To better understand the role of KH176m in the inflammatory response, we next employed a validated in vitro model system for acute inflammation: LPS activation of RAW264.7 macrophage-like mouse cells. Macrophages play a critical role in the initiation, maintenance, and resolution of inflammation and they also are a central source of PGE_2_ production^34^. Our data showed that levels of PGE_2_, PGD_2_ and 6-keto-PGF_1α_ were significantly increased after LPS stimulation in RAW264.7 cells. Similar to what was observed in human fibroblasts, KH176m selectively reduced PGE_2_ levels but did not affect the levels of PGD_2_ and 6-keto-PGF_1α_ in mouse macrophages. We hypothesized that KH176m targets mPGES-1, the inducible form of PGES that is coupled to COX-2, and functions as the terminal enzyme in LPS-induced PGE_2_ production. Indeed, we found that KH176m specifically inhibited induction of mPGES-1, without affecting the expression of COX-1, COX-2, cPGES, or mPGES-2. The inhibition of mPGES-1 was on the mRNA level leading to decreased protein levels although a direct effect on translation cannot be excluded.

Our results showed that mPGES-1 enzymatic activity was inhibited by KH176m in a dose-dependent manner, in both mouse macrophage-like cells and human fibroblasts. KH176m was found to be more potent in experiments using the rodent cells than human fibroblasts throughout our experiments. However, our studies have shown that inhibition of PGE_2_ by KH176m is a multifactorial process (involving both protein expression and enzyme inhibition). We so far have not studied these processes in enough detail to justify a direct comparison of the differ species. Earlier reports have revealed that PGE_2_ itself can drive the induction of the enzyme (mPGES-1) producing it in a so-called PGE_2_-driven positive feedback-loop^24^. We therefore hypothesized that KH176m is not only inhibiting mPGES-1 activity but as a consequence of the reduced PGE_2_ synthesis, inhibits its expression too. Indeed, by adding exogenous PGE_2_ to the cells we could counteract the effect of KH176m on mPGES1 expression, which suggests an indirect effect of KH176m on mPGES-1 expression. Additionally, we revealed that a previously known inhibitor of mPGES-1 activity, PF9184, also had an inhibitory effect on mPGES-1 expression. This effect was also inhibited by the addition of exogenous PGE_2_, which further strengthened our hypothesis.

mPGES-1 is strongly up-regulated by inflammatory stimuli and contributes to the production of pro-inflammatory, pro-nociceptive, and proangiogenic PGE_2_. Targeting mPGES-1 has recently emerged as a safer alternative to current classes of NSAIDs or Coxibs^35,36^. Both NSAIDs or Coxibs have been associated with serious cardiovascular and gastrointestinal adverse events^37^. COX-1 enzyme is constitutively expressed in most tissues and has a gastro-protective function, and inhibition of this enzyme can result in gastric damage. Although the COX-2 enzyme is mainly expressed in inflamed tissue, COX-2 selective inhibitors have been found to increase cardiovascular adverse events and are associated with an increased risk of hypertension. It has been revealed that these effects were attributed to the suppression of COX-2-mediated prostacyclin (PGI_2_) synthesis^38,39^. Indeed, PGI_2_ has been shown to play an important role in blood vessel dilation and platelet-aggregation inhibition and is cardio protective. Contrary to the upstream enzymes COX-1 and COX-2, inhibition of mPGES-1 selectively blocks inflammation-induced PGE_2_ production, without reducing the synthesis and function of other prostaglandins. Targeting mPGES-1 would therefore eliminate the adverse effects associated with the non-selective inhibition of prostaglandin synthesis by NSAIDs and Coxibs. mPGES-1 is expressed at low levels in normal tissues and upregulated in inflamed tissues and is therefore less prone to on-target adverse effects. Furthermore, recent studies have shown that mPGES-1 upregulation is involved in the pathophysiology of several inflammatory neurologic diseases, including Alzheimer’s disease, Parkinson’s disease, and glioma and in several types of cancers^7,16,17^. Therefore, inhibition of mPGES-1 by KH176m or its parent compound sonlicromanol might be of importance as alternative treatment interventions in inflammatory brain diseases and specific cancers.

In recent years, several drug discovery strategies have been employed in the identification of mPGES-1 inhibitors. The first synthetic mPGES-1 inhibitor was an indole-based carboxylic acid (MK-886), which had been earlier reported as a 5-lipoxygenase-activating protein inhibitor^40^. Studies also have revealed some endogenous fatty acids (arachidonic acid, docosahexaenoic acid) and corresponding eicosanoids such as leukotriene C4, PGJ_2_ and 15-deoxy-Δ[12,14]-PGJ_2_ as weak direct mPGES-1 inhibitors^41^. Currently, a number of diverse natural and synthetic compounds have been identified as mPGES-1 inhibitors. However, most of them have exhibited drawbacks, including high lipophilicity and interspecies differences, which has hampered preclinical evaluation of efficacy in routine animal models of inflammation. As such, only a handful of these inhibitors (such as LY3023703) have entered clinical trials^42–44^.

Safety and efficacy of sonlicromanol, the parent compound of KH176m, was evaluated in a phase 1 randomized control trial (RCT) in healthy volunteers^1^ and a Phase 2a RCT^4^ in patients with mitochondrial m.3243A > G spectrum disorder. Of importance, MELAS iPS derived endothelial cells show both pro-atherogenic and pro-inflammatory properties^45^. Our phase 1 and 2a studies revealed that sonlicromanol had an acceptable safety profile and favorable pharmacokinetics, was well tolerated over a treatment period of 28 days, and had a positive effect on cognition, an important burden for patients with mitochondrial disease. Interestingly, the pharmacokinetics analysis showed a consequent sonlicromanol to KH176m metabolism, with the maximal concentration of KH176m in plasma reaching 500 nM^1^. In the present study we show that KH176m could inhibit PGE_2_ production with IC_50_ ranging between 85 and 500 nM. We also show that PGE_2_ was elevated in cells from mitochondrial disease patients, it is therefore plausible that the effect of KH176m on PGE_2_ production plays a role in the overall mechanism of action of sonlicromanol in patients with mitochondrial disease.

In conclusion our findings show that KH176m, a metabolite found in high concentration in human subjects dosed with sonlicromanol, selectively inhibits the biosynthesis of PGE_2_ via inhibition of mPGES-1. This is of particular interest for the treatment of patients with mitochondrial diseases but may also benefit patients with other diseases associated with inflammatory pain, inflammatory neurologic diseases and inflammatory cancers.

## Methods

### Materials

KH176m is a proprietary compound developed by Khondrion (PCT/EP2016/074009)^2,3,14^. LPS from *Escherichia coli 0111:B4*, IL-1β, glutathione (GSH), iron (II) chloride (FeCl_2_), citric acid, indomethacin, and celecoxib were obtained from Sigma-Aldrich (Zwijndrecht, The Netherlands). PGH_2_ was obtained from Cayman Chemical (Hamburg, Germany). PF9184 was obtained from R&D Systems (Abingdon, United Kingdom).

### Cell culture

All primary human skin fibroblasts used throughout this study were received from RadboudUMC, Nijmegen, the Netherlands, after obtaining informed consent from donors (Supplemental Table 1). All fibroblasts used were established cell lines, so there was no direct involvement of humans and only cell lines were used. The cells were cultured in M199 (Gibco, Landsmeer, The Netherlands) containing 10% fetal bovine serum (FBS) (Greiner Bio-one, The Netherlands) and 1% penicillin/streptomycin (P/S) (Corning, Amsterdam, The Netherlands). Fibroblasts were passaged by trypsinization every 4–5 days until they reached the passage number 20, then discarded. The mouse macrophage-like cell line (RAW264.7) was purchased from Sigma-Aldrich (Zwijndrecht, The Netherlands). RAW264.7 cells were cultured in DMEM (Gibco, Landsmeer, The Netherlands) containing 10% FBS and 1% P/S. The cells were passaged by scraping every 3–4 days until they reached the passage number 20, and then discarded. All cells were maintained in a humidified atmosphere of 5% CO_2_ at 37 °C.

### Metabolomics screening

For metabolomics analysis, 150,000 fibroblasts per well were seeded to 6-well plates and cultured as described. The next day, cells were treated with KH176m (1 µM) and incubated for 24 h. Then, 1 mL culture medium was collected from each well and snap frozen in liquid nitrogen. The cells were washed with phosphate buffered saline (PBS) and detached by trypsinization. To quench the cellular metabolism, the plate was put on ice and 1 mL of ice-cold PBS was added to each well. The cell suspension (1,200 µL) was transferred to a 1.5 mL Eppendorf tube. Each aliquot was divided into a sample used for protein quantification (200 µL) and a sample used for metabolomics analyses (1 mL). Both aliquots were centrifuged (340 g, 5 min, 4 °C) and supernatants were discarded. The cell pellet for protein quantification was snap frozen in liquid nitrogen and kept at − 80 °C. The cell pellet for metabolomics analysis was washed by resuspending in 500 µL PBS, followed by centrifugation. The cell pellet was snap frozen and kept at − 80 °C.

Samples for metabolomics analysis were extracted using the validated method as described by Schoeman et al., with the following starting sample modifications^5^. Cell pellets were dissolved in 500 µL of ice cold 80% methanol in water (v:v), shaken in a bullet blender (5 min, 22 °C) and centrifuged (253,00 *g*, 5 min, 4 °C), after which 250 µL of the supernatant was collected for further analysis. The cell pellet QC pool was prepared by pooling 75 µL of the remaining sample volume of each study sample. Prior to metabolomics analyses, cell extracts were dried in a speedvac for one hour and reconstituted with 350 µL of the liquid–liquid extraction (LLE) buffer. Culture medium samples were prepared by aliquoting 350 µL medium for each study sample whereas the medium QC pool was made by pooling 150 µL from the remaining sample volume. The procedure then followed the protocol described by Schoeman et al., with samples being spiked with internal standards and antioxidant prior to a twofold LLE with butanol:ethyl acetate (1:1, v:v). The organic layers were collected and dried in a speedvac. Dried sample extracts were reconstituted and analyzed on the Shimadzu LCMS-8050 (Shimadzu, Japan) consisting of an ultra-high-performance LC (UHPLC) system connected to a triple quadrupole mass spectrometer with an ESI source. The analytes and ISTDs were measured using multiple reaction monitoring (MRMs) in either positive or negative ion mode. The data were normalized to protein concentration.

### Prostanoids assay

Cells were seeded at a density of 6000 cells/well (PHSFs) or 15,000 cells/well (RAW264.7) into 96-well plates (Greiner Bio-one, Alphen a/d Rijn, The Netherlands). After 24 h, the cells were treated with LPS (1 µg/mL) or IL-1β (1 ng/mL) in presence or absence of KH176m at the indicated concentrations and incubation times. Concentrations of PGE_2_, PGD_2_ and 6-keto-PGF_1α_ in the culture medium were determined using enzyme-linked immunosorbent assay (ELISA) kits (Enzo life, Antwerp, Belgium). Samples (100 µL) of culture medium were collected from each well and diluted with the assay buffer, if necessary. The concentration of each prostanoid was determined according to the instructions provided with the kits and interpolated from standard curves. The concentration of each prostanoid was normalized over cell number using the Calcein-AM Viability Dye (Thermo Fischer Scientific, Landsmeer, the Netherlands). Briefly, cells were incubated with 2.5 µM Calcein-AM for 30 min, then washed with DMEM (without phenol red + 10 mM HEPES); fluorescence was acquired on a FLUOstar Omega plate reader (excitation 485 nm and emission 520 nm) and analyzed with MARS-Omega data analysis software. Incubations with LPS/IL-1β and/or compounds did not systematically affect the cell viabilities.

### Measurement of PGES activity

PGES enzyme activities in cell membranes were measured by quantifying the conversion of PGH_2_ to PGE_2_ using a modified protocol of a previously described method^17^. Briefly, cells were stimulated with LPS (1 µg/mL) for 24 h, and collected and isolated by sonication (10 s, three times at 1 min intervals) in 300 µL ice-cold 1 M Tris–HCl, pH 8.0. After centrifugation at 15,000 *g* for 10 min at 4 °C, the supernatant was collected and the microsomal membrane fraction was pelleted by further centrifugation a 100,000 g for 1 h at 4 °C. The pellets were resuspended in 100 µL 0.1 M Tris–HCl, pH 8.0, containing protease inhibitors (cOmplete ULTRA Tablets, Mini, EDTA-free, EASYpack Protease Inhibitor Cocktail, from Roche, Woerden, The Netherlands), and were used as enzyme source to measure PGES activity.

Briefly, the protein content of the microsomal membrane fractions was quantified using a Bradford assay. For each incubation, the volume corresponding to 90 µg of total protein was mixed with test compounds (KH176m or PF9184) in 0.1 M Tris–HCl, pH 8.0 containing 2.5 mM GSH and 14 µM indomethacin in a final volume of 120 μL and incubated for 15 min at room temperature to allow interaction with mPGES-1. Activity measurements were initiated by the addition of PGH_2_ (2 µg). After incubation on ice for 60 s, the reaction was stopped by via 40 mM FeCl_2_ solution containing 80 mM citric acid in PGE_2_ ELISA assay buffer. The PGE_2_ concentrations in the samples were subsequently quantified using a PGE_2_ ELISA kit as described above.

### COX enzymatic activity-cell free assay

The activity of KH176m on COX-1 and COX-2 was determined using COX Inhibitor Screening Kit (Bio-Vision, Huissen, The Netherlands) following the manufacturer's instructions.

### Western blot analysis

Cells were lysed in buffer (50 mM Tris–HCl pH8.0, 150 mM NaCl, 0.2% Triton X100, containing 0.1 mg/mL DNAse (Sigma-Aldrich, Zwijndrecht, The Netherlands) with protease inhibitor (cOmplete ULTRA Tablets, Mini, EDTA-free, EASYpack Protease Inhibitor Cocktail) and PhosStop (Phosphatase inhibitor) from Roche (Woerden, The Netherlands). Total proteins (45 µg) were separated by 10% or 12% sodium dodecyl sulfate–polyacrylamide gel electrophoresis (SDS-PAGE) and transferred to a polyvinylidene difluoride (PVDF) membrane (Merck Millipore, Amsterdam, The Netherlands). Membranes were blocked with 5% BSA in TBST (Tris Buffered Saline with 0.1% Tween 20) for 1 h at room temperature and then incubated overnight with primary antibodies at 4 °C (primary antibodies are listed in Supplemental Table 2). Corresponding secondary antibodies (Goat anti Mouse IRDye 680 or Goat anti Rabbit IRDye 800, 1:10,000, Odyssey, Leusden, The Netherlands) were used to detect the primary antibodies. Finally, membranes were scanned and analyzed on the Odyssey CLx Infrared Imaging System (LI-COR, Lincoln, The United States).

### RNA extraction and qRT-PCR

Total RNA was isolated from cells using the TRIzol reagent (Invitrogen, Uden, The Netherlands). The obtained mRNA was reverse-transcribed to cDNA from 2 µg of total RNA using a FirstStrand cDNA Synthesis Kit (Roche, Woerden, The Netherlands). Quantitative PCR analysis was performed in a total volume of 20 µL containing cDNA template, sense and antisense primers, and SYBR Green master mix (QIAGEN, Venlo, The Netherlands). Data was expressed as fold changes relative to control conditions (unstimulated cells) normalized to housekeeping gene PPIA using the ^∆∆^CT method^46^. Each PCR was performed in duplicate at two different time points during three independent experiments (primer information is shown in Supplemental Table 3).

### Statistical assay

Unless otherwise indicated, all experiments were performed with three independent biological repeats with each three technical repeats. The results were presented as mean ± S.D. Statistical analysis was performed with GraphPad Prism (GraphPad Prism 7.0 Software). Experiments were designed to compare multiple groups were determined by analysis of variance (ANOVA). Experiments were designed to determine whether the effects of stress were dependent on vehicle conditions. Variance between the experimental groups was determined by Student *t-test*. *p* < *0.05* was considered statistically significant. Information about the number of samples (n) is included in the figures and figure legends.

## Supplementary Information

Supplementary Information.

## Data Availability

The data that support the finding of this study are available on the request from the corresponding author [H.R.].
