# Supplementary material for: Mechanism of action and potential applications of selective inhibition of microsomal prostaglandin E synthase-1-mediated PGE2 biosynthesis by sonlicromanol’s metabolite KH176m
Source: Sci Rep. 2021 Jan 13;11:880. doi: 10.1038/s41598-020-79466-w (PMC7806836; doi:10.1038/s41598-020-79466-w)
Supplement: Supplementary file 1 — Supplementary Information. [file 41598_2020_79466_MOESM1_ESM.docx]

**Mechanism of action and potential applications of selective inhibition of microsomal prostaglandin E synthase-1-mediated PGE2 biosynthesis by sonlicromanol’s metabolite KH176m**

Jiang X.^1,2^, Renkema H.^1 *^, Pennings B. ^1^, Pecheritsyna S.^1^, Schoeman J.C.^3^, Hankemeier T.^3^, Smeitink J.^1,2^, Beyrath J.^1^

1. Khondrion BV, Nijmegen, The Netherlands.

2. Department of Pediatrics, RCMM, RadboudUMC, Nijmegen, The Netherlands.

3. Faculty of Science, Leiden Academic Centre for Drug Research, Analytical BioSciences, Einsteinweg 55, 2333 CC, Leiden, The Netherlands.

Author List:

Xiaolan Jiang [Jiang@khondrion.com](mailto:Jiang@khondrion.com)

Herma Renkema [Renkema@khondrion.com](mailto:Renkema@khondrion.com)

Bas Pennings [Pennings@khondrion.com](mailto:Pennings@khondrion.com)

Svetlana Pecheritsyna [Pecheritsyna@khondrion.com](mailto:Pecheritsyna@khondrion.com)

Johannes Cornelius Schoeman [nelus_schoeman@outlook.com](mailto:nelus_schoeman@outlook.com)

Thomas Hankemeier [hankemeier@lacdr.leidenuniv.nl](mailto:hankemeier@lacdr.leidenuniv.nl)

Jan Smeitink Smeitink@khondrion.com

Julien Beyrath Beyrath@khondrion.com

| **Cell line** | **Genome** | **Affected OXPHOS Complex** | **Gender** |
| --- | --- | --- | --- |
| **C5120** | **-** | **-** | **M** |
| **C5119** | **-** | **-** | **F** |
| **C5118** | **-** | **-** | **F** |
| **S7-5175** | **Nuclear** | **Complex I** | **M** |
| **S2-7277** | **Nuclear** | **Complex I** | **F** |
| **V1-5171** | **Nuclear** | **Complex I** | **M** |

**Supplemental Table 1. Detailed information on the human fibroblast cell lines used in the study.**

| **Name** | **Company** | **Host** | **Dilution** | **Protein Size** |
| --- | --- | --- | --- | --- |
| **COX-1** | RD systems | Mouse | 1:250 | 70KD |
| **COX-2** | ThermoFisher Scientific | Rabbit | 1:500 | 70KD |
| **mPGES-1** | Agrisera | Rabbit | 1:5,000 | 16KD |
| **mPGES-2** | Cayman | Rabbit | 1:200 | 33KD |
| **cPGES** | Cayman | Rabbit | 1:200 | 23KD |
| **β-actin** | Sigma-Aldrich | Mouse | 1:10,000 | 42KD |

**Supplemental Table 2: Antibodies used for western blot analysis**

| **Target Gene** | **Organism** | **Primers (5’ 3’)** |
| --- | --- | --- |
| **COX-1** | Mouse | Forward: GATTGTACTCGCACGGGCTAC |
|  |  | Reverse: GGATAAGGTTGGACCGCACT |
| **COX-2** | Mouse | Forward: AGGACTCTGCTCACGAAGGA |
|  |  | Reverse: TGACATGGATTGGAACAGCA |
| **mPGES-1** | Mouse | Forward: AGCA CACTGCTGGTCATCAA |
|  |  | Reverse: CTCCACATCTGGGTCACTCC |
| **mPGES-2** | Mouse | Forward: GCTGGGGCTGTACCACAC |
|  |  | Reverse: GATTCACCTCCACCACCTGA |
| **cPGES** | Mouse | Forward: GGTAGAGACCGCCGGAGT |
|  |  | Reverse: TCGTACCACTTTGCAGAAGCA |
| **PPIA** | Mouse | Forward: AGGGTGGTGACTTTACACGC |
|  |  | Reverse: GATGCCAGGACCTGTATGCT |
| **mPGES-1** | Human | Forward: AGTATTGCAGGAGCGACC |
|  |  | Reverse: CCAGAAAGGAGTAGACGAAGC |
| **GAPDH** | Human | Forward: TGCACCACCAACTGCTTAGC |
|  |  | Reverse: GGCATGGACTGTGGTCATGAG |

**Supplemental Table 3: Primers used for qRT-PCR**

**
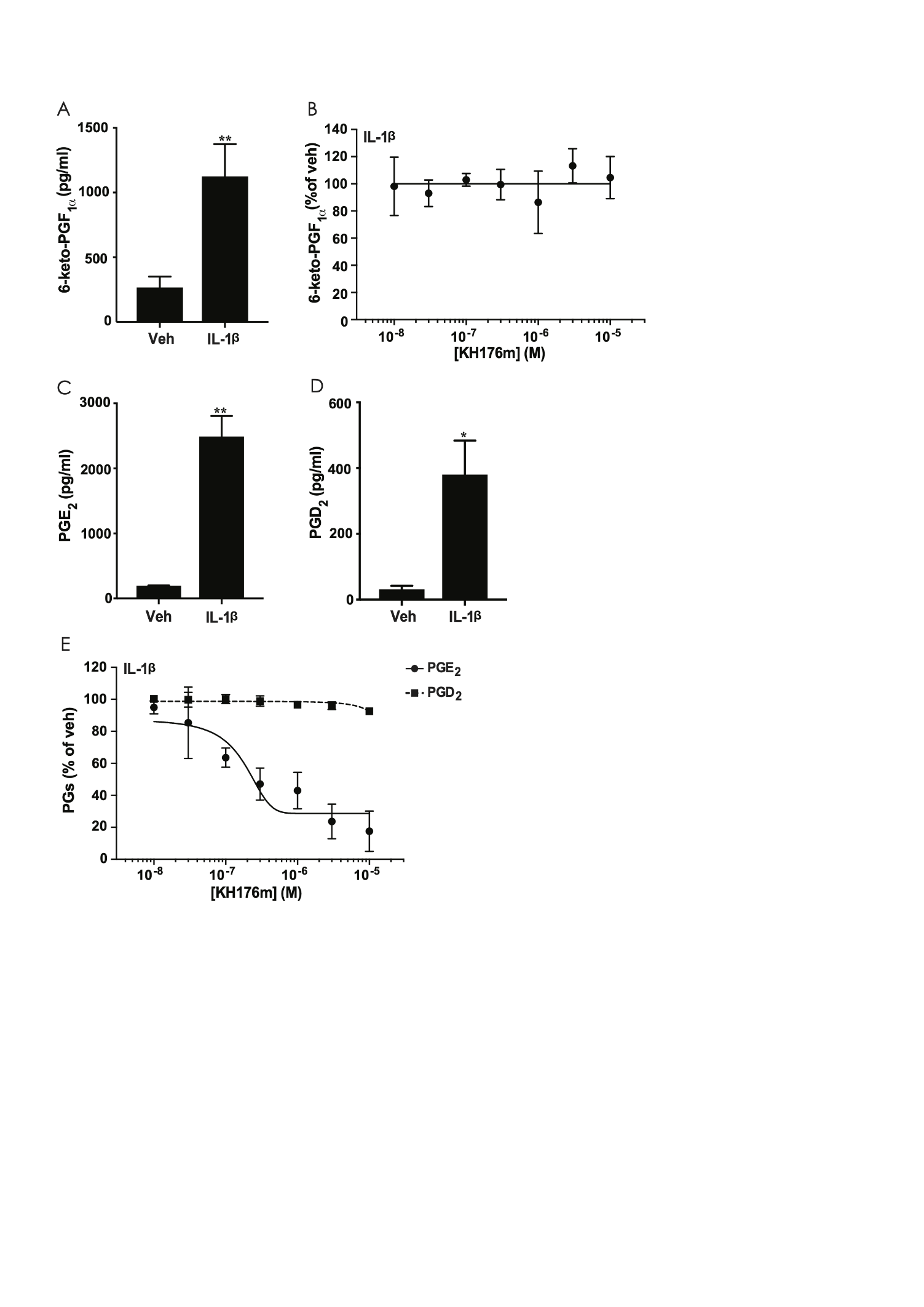
**

**Supplemental Figure 1. KH176m has no effect on 6-keto-PGF_1α_ production in human fibroblast and PGD_2_ production in RAW264.7 cells.** Level of 6-keto-PGF_1α_ was analyzed in culture medium of human fibroblasts stimulated with IL-1β (1 ng/mL) for 24 h alone (A) or in combination with increasing concentrations of KH176m (B) (n=3). Levels of PGE_2_ and PGD_2_ were analyzed in culture medium of RAW264.7 cells stimulated with IL-1β (1 ng/mL) for 24 h alone (C and D) or in combination with increasing concentrations of KH176m (E) (IL-1β alone set as 100%) (n=3). **, p<0.05; **, p<0.005; ***, p<0.001*; significant differences compared with vehicles.


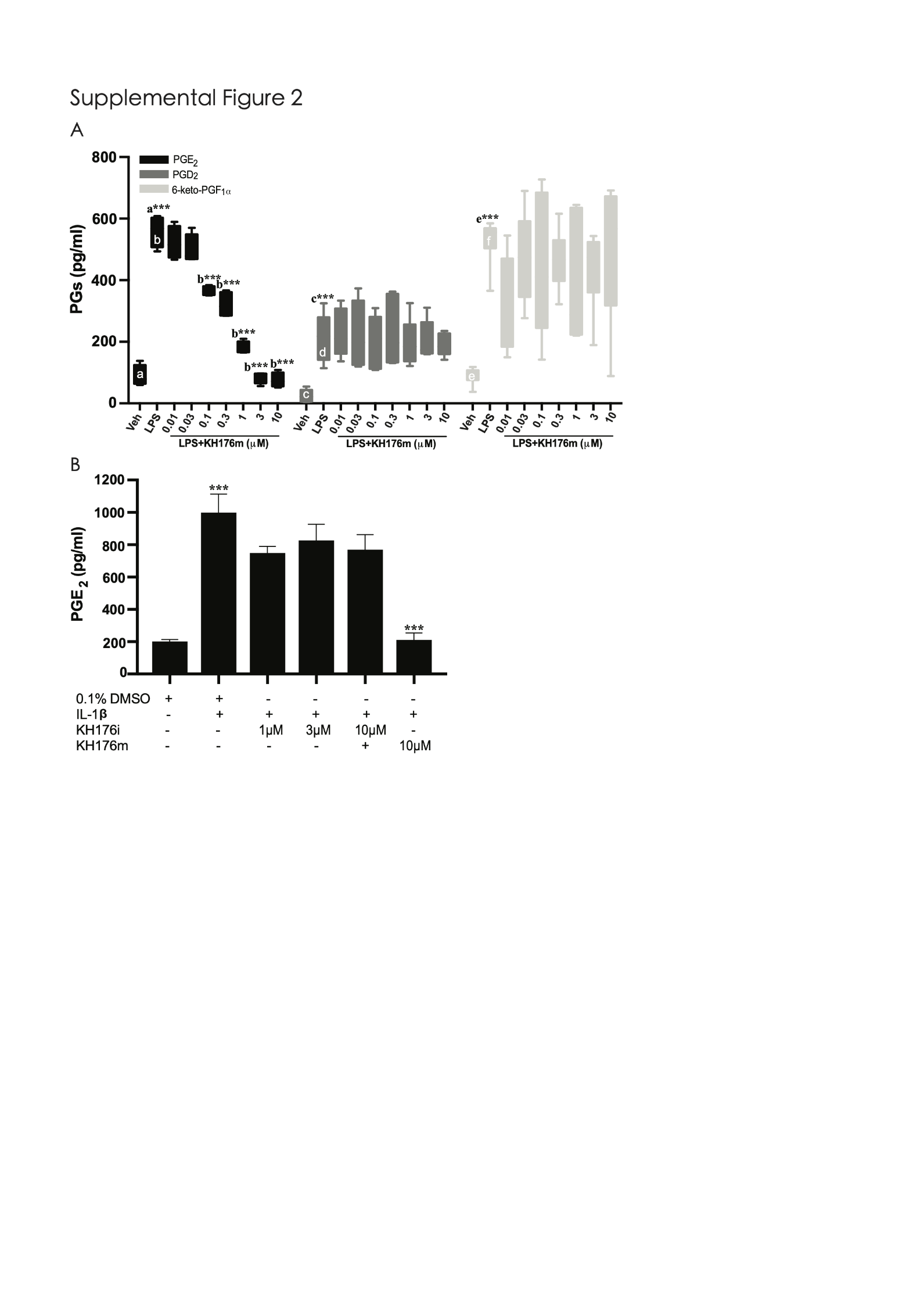


**Supplemental Figure 2. KH176m has no effect on PGD_2_ and 6-keto-PGF_1α_ production.** (A) Levels of PGE_2_, PGD_2_, and 6-keto-PGF_1α_ were analyzed in culture medium of RAW264.7 cells stimulated with LPS (1 µg/mL) for 24 h alone or in combination with increasing concentrations of KH176m (n=3) **, p<0.05; **, p<0.005; ***, p<0.001*; significant differences compared with the marked conditions (a,b,c,d,e,f). (B) Levels of PGE_2_ was analyzed in culture medium of C20 fibroblasts stimulated with IL-1β (1 ng/mL) for 24 h alone or in combination with increasing concentrations of KH176i or KH176m (n=3) ****, p<0.001*; significant difference compared with vehicles.


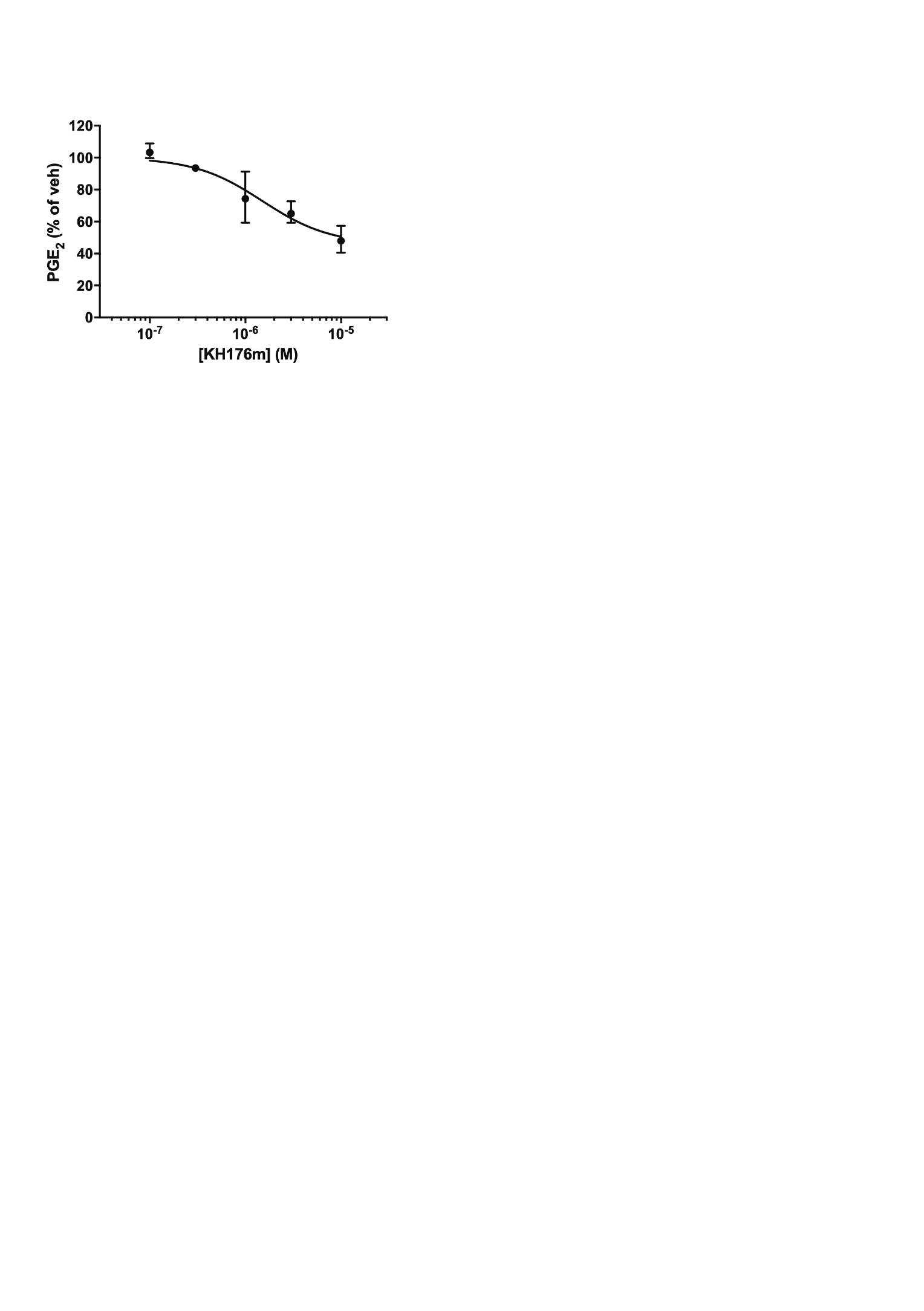


**Supplemental Figure 3: KH176m blocks mPGES-1 activity in human fibroblasts.** Fibroblasts were treated with IL-1β (1 ng/mL) for 24 h and microsomes were isolated and used as source of mPGES-1. mPGES-1 activity was measured in microsomes fraction as the conversion of PGH_2_ to PGE_2_ (n=3).

**
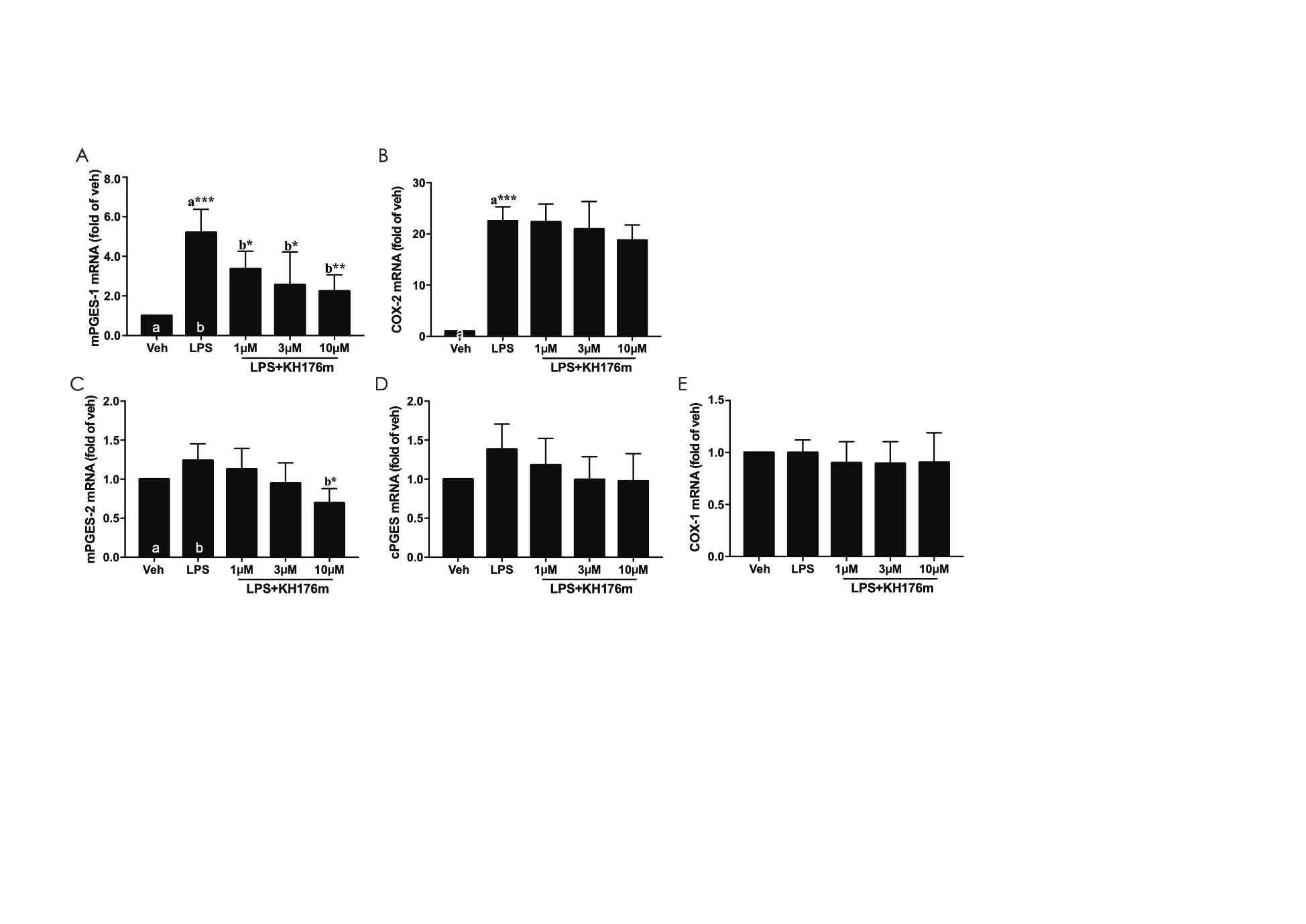
**

**Supplemental Figure 4. KH176m selectively decreases the expression of mPGES-1 enzyme.** RAW264.7 cells were treated with various concentrations of KH176m in the presence of LPS (1 µg/mL) for 6 h. (A-E) Gene expression was analyzed by qRT-PCR for (A) mPGES-1, (B) COX-2, (C) mPGES-2, (D) cPGES, and (E) COX-1 (n=3) **, p<0.05; **, p<0.005; ***, p<0.001*; significant differences compared with the marked conditions (a,b).


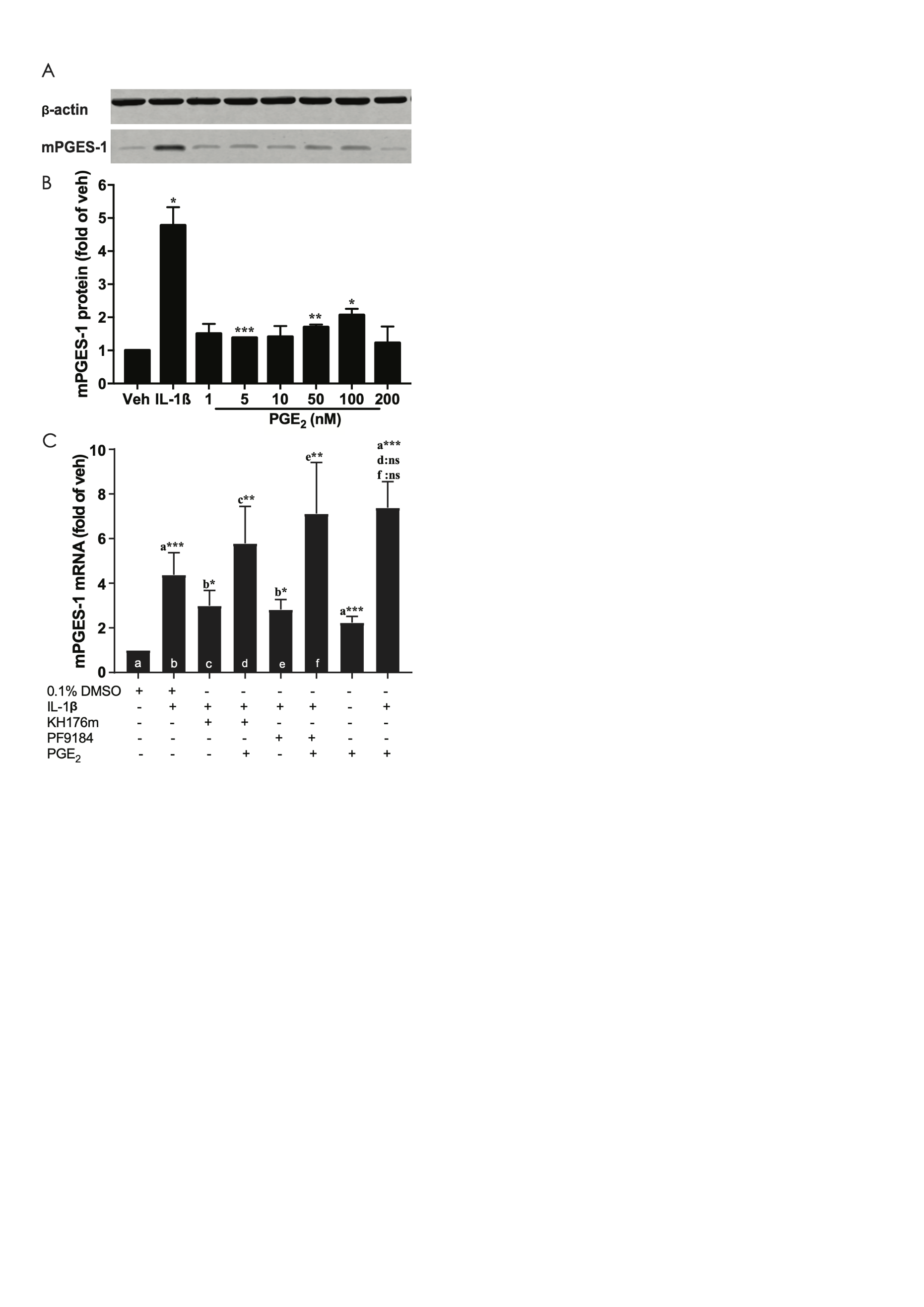


**Supplemental Figure 5: Exogenous PGE_2_ reversed the effect of KH176m in IL-1β stimulated fibroblasts.** Fibroblasts were treated with IL-1β (1 ng/mL) or PGE_2_ (100 nM) for 24 h. (A) Protein was isolated and separated by SDS-PAGE; expression levels of indicated proteins were analyzed by western blot analysis. (B) Quantification of the western blot analysis of panel A (n=3). (C) Gene expression was analyzed by qRT-PCR (n=3). **, p<0.05; **, p<0.005; ***, p<0.001*; significant differences compared with the marked conditions (a,b,c,d,e).

**Full-length blot**

**Figure 2**

**mPGES-1 and actin**

**
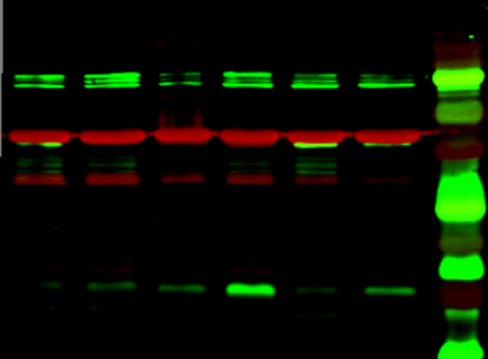
**

mPGES-1

actin

cc

cc

**Full-length blot**

**Figure 6**

**mPGES-1 and actin**

**
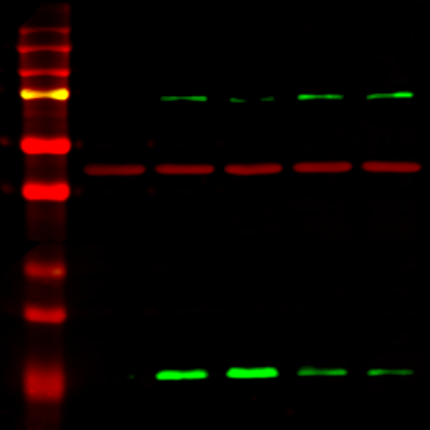
**

mPGES-1

actin

**mPGES-2 and actin**

**
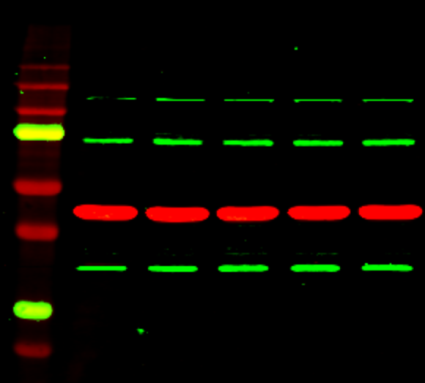
**

mPGES-2

actin

c

c

**cPGES and actin**

**
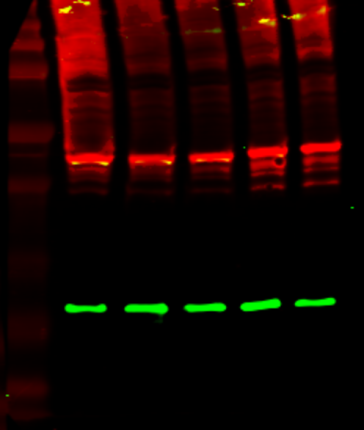
**

cPGES

actin

c

c

**COX1 and actin**

**
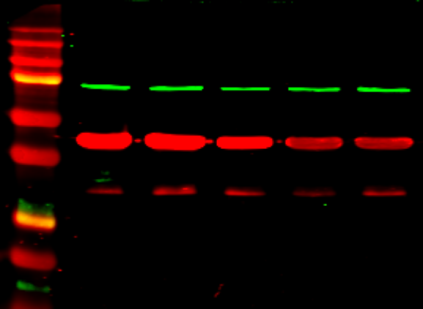
**

actin

COX1

cc

cc

**COX2 and actin**

**
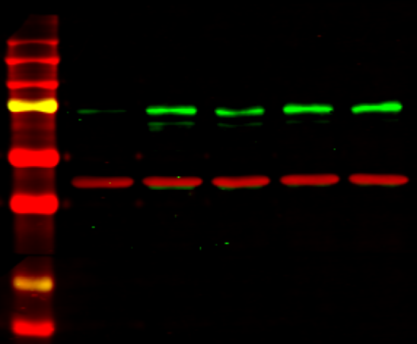
**

COX2

actin

cc

cc

**Full-length blot Figure 7 (blot was cut to test COX2 expression at the same time)**

**
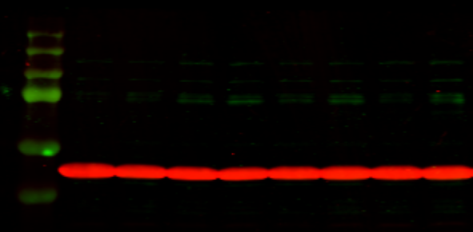
**

COX2

cc

cc

actin

**
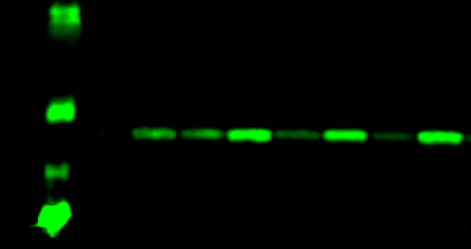
**

mPGES-1

cc

**Full-length blot Supplemental Figure 5**

**mPGES-1 and actin**

**
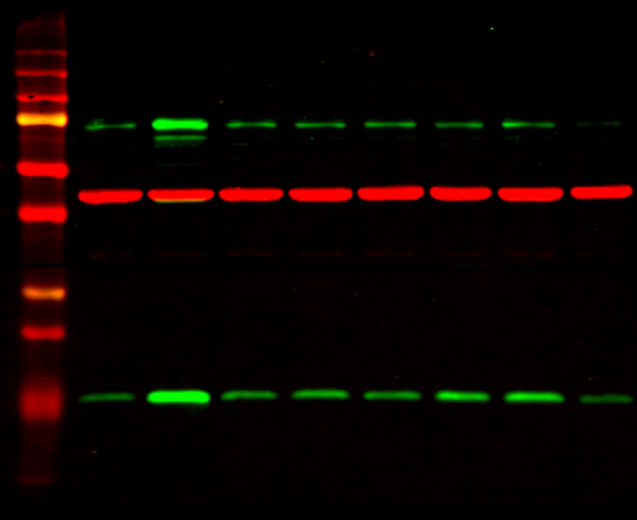
**

mPGES-1

cc

cc

actin
